# Weight management in dogs and cats

**DOI:** 10.1186/1751-0147-57-S1-K7

**Published:** 2015-09-25

**Authors:** Alexander J German

**Affiliations:** 1 Institute of Ageing and Chronic Disease, University of Liverpool, Liverpool, UK

## 

Obesity is defined as an accumulation of excessive amounts of adipose tissue in the body, and predisposes to a variety of diseases including diabetes mellitus, and osteoarthritis. In most animals, obesity is the result of a simple imbalance between energy intake and energy expenditure. Therefore, at its simplest, successful management of obesity usually involves reversing this imbalance by reducing energy intake and/or increasing energy expenditure. This lecture will discuss current thoughts on both conventional and novel treatments for of obesity in dogs and cats, and stimulate discussion on factors required for long-term success.

